# Landscape of Spinal Muscular Atrophy Newborn Screening in the United States: 2018–2021

**DOI:** 10.3390/ijns7030033

**Published:** 2021-06-24

**Authors:** Kshea Hale, Jelili Ojodu, Sikha Singh

**Affiliations:** Association of Public Health Laboratories, Silver Spring, MD 20910, USA; jelili.ojodu@aphl.org (J.O.); sikha.singh@aphl.org (S.S.)

**Keywords:** newborn screening, spinal muscular atrophy, new disorders implementation

## Abstract

Newborn screening (NBS) programs identify newborns at increased risk for genetic disorders, linking these newborns to timely intervention and potentially life-saving treatment. In the United States, the Health and Human Services (HHS) Advisory Committee on Heritable Disorders in Newborns and Children (ACHDNC) recommends the disorders for state NBS programs to screen. ACHDNC updated the Recommended Uniform Screening Panel to include Spinal Muscular Atrophy (SMA) in July 2018. As of June 2021, 34 state NBS programs had fully implemented SMA newborn screening, and at least 8 programs were pursuing implementation. This article will review current SMA screening processes, considerations, challenges, and status.

## 1. Introduction

Newborn screening (NBS) is a state-implemented public health program intended to identify newborns at increased risk of certain genetic disorders. Some disorders, if undetected and left untreated or detected later within the life course, might cause mild to severe disability and/or premature death for the individual. Through the timely implementation of the newborn screening process, infants detected with these disorders may receive early interventions, potentially reducing short-and long-term adverse health outcomes and improving their overall quality of life. The Health and Human Services (HHS) Advisory Committee on Heritable Disorders in Newborns and Children (ACHDNC) recommends the disorders for state NBS programs to screen [1]. The approved disorders are included on the Recommended Uniform Screening Panel (RUSP), with state newborn screening programs screening for at least 30 of 35 core disorders on the RUSP as of June 2021 [2]. The RUSP was updated to include Spinal Muscular Atrophy (SMA) in July 2018.

Approximately 1 in every 11,000 babies are born with SMA in the United States each year [3]. However, the incidence rate varies by state as established during the evidence review process during the deliberation of whether to add SMA to the RUSP [4]. Initial screening studies in New York revealed a lower birth prevalence (1 in 21,000 as of February 2020) than the published 1 in 11,000 estimated from clinical detection [5]. Globally, few and small studies have been performed, with estimated incidence of SMA in pan-Europe as 1 in 3900 to 1 in 16,000 [6]. As additional global screening results are made available, surveillance estimates will become more precise. The Association of Public Health Laboratories’ (APHL) NewSTEPs Data Repository collects information on confirmed cases detected by newborn screening in the US for all disorders annually. They report that at least 45 cases of SMA were detected by newborn screening for the years 2018-2021, with 34 states offering population screening as of June 2021.

As one of the leading genetic causes of mortality for children under the age of 2 years, SMA is a group of genetic disorders that affects the motor neurons in the body. It is primarily caused by defects or mutations in the survival motor neuron 1 *(SMN 1)* gene. The *SMN 1* gene provides instructions for producing the survival motor neuron (SMN) protein, which is essential for the maintenance of the motor neurons located in the spinal cord and brainstem [7]. Motor neurons control essential voluntary muscular movement such as crawling, walking, chewing, swallowing, speaking, and breathing. SMA results in the loss of those motor neurons, leading to progressive muscle weakness and atrophy.

The exact course of the disorder and severity of symptoms differs by SMA type and age of onset. There are five types of SMA. Type 0 and type 1 are the most severe and may result in death in early infancy [8]. Individuals with type 2 may survive into adolescence or early adulthood, and those diagnosed with type 3 or type 4 may have a normal life expectancy [9]. Early risk assessment through newborn screening is important to improve long-term outcomes. When SMA is diagnosed and treated early, it is possible for patients to achieve motor developmental milestones [10].

## 2. SMA Screening Method

The exon 7 deletion in the *SMN1* gene identifies approximately 95% of newborns with SMA. All (*n* = 34) newborn screening programs currently screening for SMA utilize a real-time PCR (qPCR) first-tier methodology to detect the deletion of exon 7 in *SMN1,* as seen in Table 1, and further elaborated in Appendix A. Some programs implement a digital drop PCR or a reverse-transcriptase (RT) PCR second-tier methodology to determine the *SMN2* copy number as well (*n* = 8), while other programs repeat the first-tier methodology on the original dried blood spot on the next day (a confirmation duplicate). Five states offer a three-tier, high-throughput screening algorithm [11]. Not all programs implement a second-tier screen, reflexing the newborn screening risk assessment results to a clinical laboratory. Screening algorithms are determined at the state level, taking into consideration feasibility of the screen, demographics, and laboratory capabilities. A majority of NBS programs (*n* = 25) currently screening for SMA also multiplex the screening of SMA with severe combined immunodeficiency (SCID), a disorder that was added to the RUSP in 2010, and that all states have been screening for since at least 2018 [12].

## 3. SMA Implementation Process and Screening Considerations

**Figure 1:**
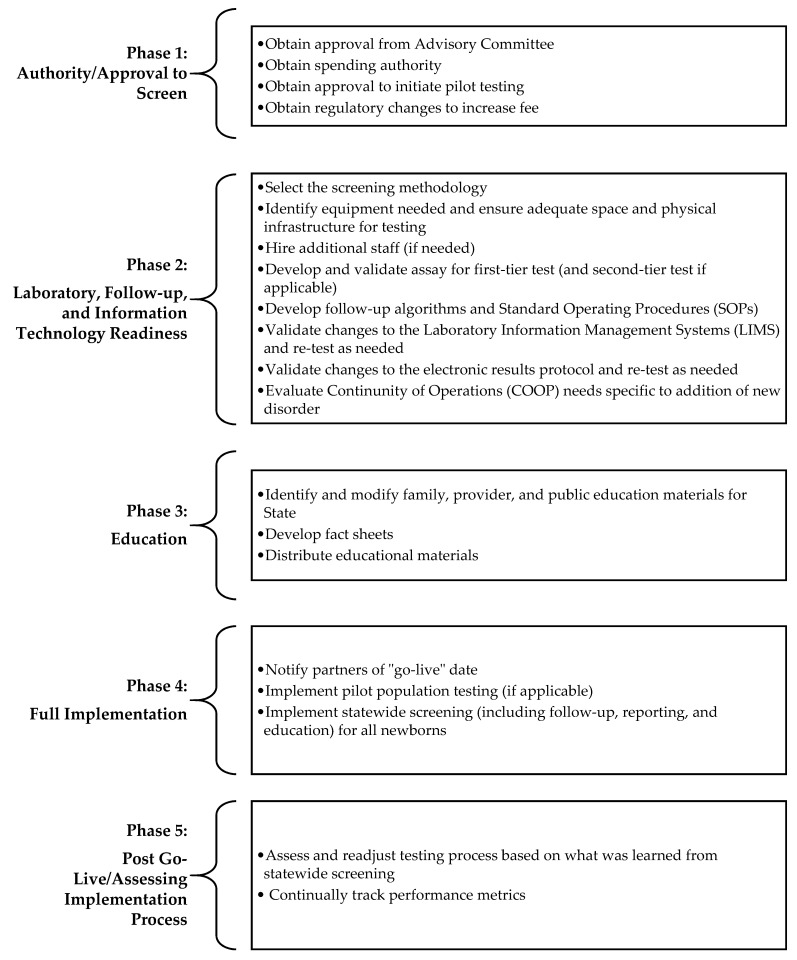
Phases of implementation for new disorders in newborn screening [13].

When pursuing full population screening of SMA, there are several phases of implementation, which vary by state, that NBS programs must complete and that impact how long a state may take to achieve population screening. A breakdown of these phases is found in Figure 1. While many of the key activities such as developing a budget, purchasing equipment, and integrating testing into current workflow will occur across all states, the duration and burden of these activities are dependent on parameters such as the size of a state, demographics, hours of operation, fees, number of screens performed, methodologies used for screening, etc. For example, while many programs increase the newborn screening fee when adding disorders to their state panels, others do not.

As newborn screening programs prepare for and enter into the second phase (laboratory, follow-up, and information technology readiness) of implementation, there are several factors NBS programs need to consider to ensure readiness to screen for SMA. A summary of SMA laboratory and follow-up screening considerations and questions is elaborated in Table 2.

## 4. State of SMA NBS Implementation

**Figure 2:**
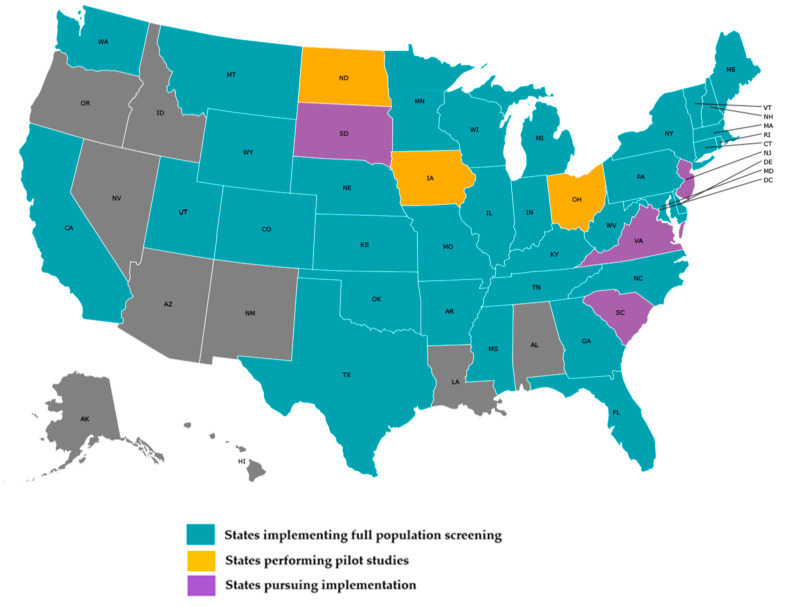
State NBS programs screening for SMA, as of 8 June 2021.

As of 8 June 2021, 34 state NBS programs are offering population-wide screening for SMA, as seen in Figure 2. This number does not include states conducting pilot testing, frequently performed in states prior to full implementation to validate the screening assay and to evaluate and refine results reporting and communication mechanisms [14]. A least seven additional programs are pursuing implementation for SMA as of June 2021. Approximately 82% of the 34 NBS programs screening for SMA implemented full population screening within two years of its addition to the RUSP. The number of NBS programs screening for this disorder surpasses the 29 NBS programs currently screening for Pompe disease, which was added to the RUSP in 2015, and the 27 NBS programs screening for mucopolysaccharidosis type I (MPS I) and the 22 newborn screening programs screening for X-linked adrenoleukodystrophy (X-ALD), which were both added in 2016. As indicated in Table 3, the average time to fully implement SMA screening is approximately 24 months, Pompe and MPS I are 40 and 37 months respectively, and X-ALD is 28 months. The most time-intensive activities for each disorder are listed in Table 4.

The table includes NBS programs that selected “complete” for full implementation. Time is calculated from earliest start date entered by NBS program to the implementation date for statewide screening. Programs that did not provide dates are excluded.

Several state NBS programs have noted that SMA is a relatively simple screen to implement since testing for the presence or absence of a gene yields minimal false-positive results, reducing the need to include a second-tier test [15]. When implementing screening for SMA, most NBS programs multiplexed SMA with SCID (Table 1), the benefit of which is that minimal laboratory staffing and technology changes are needed, and the state may not require a significant fee increase to include SMA on their panels, as compared with other newer disorders that require more complex laboratory, information technology, follow-up, and education considerations [16].

To support state NBS programs as they implemented screening for SMA, the Association of Public Health Laboratories (APHL) Newborn Screening Technical assistance and Evaluation Program (NewSTEPs) provided funding and educational opportunities through a cooperative agreement from the Health Resources and Services Administration (HRSA). State NBS programs utilized funding for SMA screening implementation from NewSTEPs to purchase equipment and supplies, to travel to other state newborn screening laboratories for training, to hire staff, to develop educational resources, and to perform software modifications. State NBS programs applied for funding through a competitive and goals-driven request for proposals (RFP) process. State NBS programs also received direct funding from the Centers for Disease Control and Prevention (CDC) and other newborn screening stakeholders and partners to purchase equipment and supplies, hire staff, and complete other implementation activities.

Programs receiving NewSTEPs funding achieved the following milestones during the funding period: completed on-site training with PerkinElmer to use Janus G3 Mini and Quant Studio DX instruments; received training on Evoya LIMS software; developed a validation plan, completed SMA-SCID validation and test implementation; procured and validated QuantStudio and Pro real-time PCR instruments; added the SMA screening instrument to the LIMS for testing and reporting; identified and procured needed Perkin Elmer SpecimenGate software modifications for SMA implementation; and developed educational materials. To address educational needs, APHL hosted two national webinar series in 2018 and 2019 to discuss SMA implementation with subject matter experts and to support states as they prepare for screening. Speakers discussed the following: SMA screening methodologies and follow-up requirements and processes, state educational strategies, current approved FDA treatments, long-term implications and outcomes of treatment, and physician experiences treating SMA using Spinraza and Zolgensma.

## 5. Challenges

Despite SMA screening being relatively simpler to implement than the other newer disorders added to the RUSP, the timing of implementation in 2020 for the five APHL NewSTEPs funded states coincided with the COVID-19 pandemic, and this confluence of events resulted in several challenges. The specific challenges as described by the funded programs to NewSTEPs via routine discussions and teleconference check-ins are described in Table 5.

## 6. Lessons Learned and Recommendations

States preparing to screen for SMA should consider the program’s capacity and testing needs and begin planning as early as possible to enhance capacity to screen. Planning should include staffing needs, spacing requirements, reagent needs, and instrument capacity to meet SMA screening requirements.

When implementing a new disorder during a pandemic, state NBS programs receiving NewSTEPs funding recommend NBS programs perform the steps listed in Table 6.

## 7. Conclusions

The ACHDNC updated the RUSP to include SMA in July 2018. To expand and facilitate screening for SMA, the CDC, APHL NewSTEPs through funding from the Health Resources and Services Administration (HRSA), and other NBS stakeholders provided funding and educational opportunities to US newborn screening programs. Through these funding opportunities, state NBS programs purchased the equipment and supplies needed to perform screening. To provide further support to state programs, APHL hosted several webinars to discuss laboratory, follow-up, and treatment considerations for SMA NBS screening.

As of June 2021, 34 states have implemented SMA screening, despite challenges and delays due to the COVID-19 pandemic. To assist the remaining states that intend to implement SMA screening, APHL will, through its HRSA-funded NewSTEPs program, continue to provide technical support, educational resources, and funding assistance. APHL will also continue to collect, collate, analyze, and disseminate challenges, successes, and lessons learned during the implementation process in an effort to enable continuous quality improvements and to strengthen the body of data around the national landscape for SMA newborn screening.

## Figures and Tables

**Table 1:** Summary of spinal muscular atrophy newborn screening methodologies in the United States, June 2021 (state specific details in Appendix A).

SMA Screening Methodologies	# of States
Multiplex with SCID	25
First-tier SMA screen using teal-time PCR for detection of homozygous deletion of exon 7 in *SMN1*	34
First- and second-tier SMA screen	8
First-, second-, and third-tier SMA screen	5

**Table 2:** Laboratory and follow-up screening considerations and questions for SMA.

Laboratory Considerations
What screening method should be utilized? Single-plex or multiplex Single or multi-tier assays
What will be the cutoff value and reference gene?
What reference materials to use in order to calibrate instrumentation?
What material should be utilized for daily positive quality control (QC), and from where do you obtain it?
Should the *SMN2* copy number be determined within the screening lab? If yes, why and by what method?
How will you validate your Laboratory Information Management Systems (LIMS)
Considerations for multiplexing with SCID:Platforms used Cut-off value and validation plan What to do in the case if screening fails for one disorder, but not the other? Is there an impact on re-test rate when multiplexing?
**Follow-up Program Considerations**
What are the reporting and follow-up guidelines?
What follow-up data should be collected? Both upon diagnosis and after diagnosis? Lab results? Clinical findings? Other?
What does surveillance look like for SMA?
How to interpret results of multiplexed assays?
What specialists need to be involved in the follow-up process?
Are specialists determining SMA type based on *SMN2* copy numbers and clinical symptoms present at diagnosis?
What wording should be used for the interpretation of normal results/positive results on the NBS report?
Specialist involvement: Are cases going directly to neurologists or are they filtered through geneticists? How are positive results reported? To a primary care physician? To a specialist? Both? Are results called out on weekends?
What is the referral rate? How many (or what % of) released results to primary care physician/specialist yielded an SMA diagnosis?

**Table 3:** Time to full implementation of statewide screening for new disorders. Preliminary results from the NewSTEPs New Disorder Readiness Scale, as of 31 August 2019.

Disorder	No. of States	Mean (Months)	Median (Months)	Min (Months)	Max (Months)	Range (Months)
Pompe	11	39.64	28	13	99	86
MPS I	13	36.92	28	13	75	62
X-ALD	8	27.63	30.5	16	36	20
SMA	5	24.40	20	17	38	21

**Table 4:** The most time-intensive activity by disorder. Preliminary results from NewSTEPs New Disorder Readiness Scale, as of 31 August 2019.

Disorder	Activity	Average Time (Months)
Pompe	Develop and gain buy-in for STFU protocols for abnormal screens	15.13
MPS I	Identify screening methodology/assay for first-tier testing	14.80
X-ALD	Develop/validate assay for second-tier testing	19.67
SMA	Obtain approval from the state budget authority	10

**Table 5:** Challenges reported by state newborn screening programs receiving NewSTEPs funding to implement SMA newborn screening coinciding with the 2020 global COVID-19 pandemic.

Challenges	Description
Competing Priorities/Shift of Duties	Several states experienced delays as a result of the COVID-19 pandemic. NBS program staff from all areas shifted focus from routine duties to address the crisis, including providing testing for COVID-19. This affected NBS staff, fiscal/accounting staff, and other support staff in public health laboratories.
Procurement of Lab Supplies	The COVID-19 pandemic affected the ability to procure lab supplies needed for SMA screening, such as pipette tips and reagents.
External Partners	The COVID-19 pandemic impacted staffing situations with external partners.
Legislative Delays	The COVID-19 pandemic delayed the legislative process, resulting in a delay in the approval and implementation of SMA screening.
Malfunctioning of Instrumentation	One program experienced problems with liquid handling instruments. The instruments were not functioning correctly due to manufacturing errors. The program reported that one system had to be replaced and the other modified. The manufacturer was responsive and spent several days working onsite to fix the issue. This issue took approximately six weeks to resolve.

**Table 6:** Recommendations for implementing screening for a new disorder during a pandemic.

Steps	Recommendation
Step 1	Order supplies needed with a longer lead time to ensure that manufacturing and transportation delays do not negatively impact validation timelines.
Step 2	Continue to meet with implementation team composed of laboratory leadership, technical experts, follow-up leadership, and informatics support staff to ensure that all parties are informed of validation and regulatory progress.
Step 3	Hire additional staff whenever possible before staffing shortages force timeline delays.

## Data Availability

No new data were created or analyzed in the study. Data sharing is not applicable to this article.

## Appendix A

**Table A1:** State Screening Methods and Targets for SMA Newborn Screening.

**States Offering Population NBS for SMA as of June 2021 ***	**First-Tier Method**	**First-Tier Target**	**Second-Tier Method ^**	**Second-Tier Target**	**Multiplex with SCID**
Arkansas	qPCR	*SMN1*	None	N/A	Yes
California	qPCR	*SMN1*	None	N/A	No
Colorado	qPCR	*SMN1*	None	N/A	Yes
Connecticut	qPCR	*SMN1*	None	N/A	Yes
Delaware	qPCR	*SMN1*	Multiplex Ligation PCR Amplification (MLPA)	*SMN1and SMN2*	No
Florida	qPCR	*SMN1*	None	N/A	Yes
Georgia	qPCR	*SMN1*	None	N/A	Yes
Illinois	qPCR	*SMN1*	None	N/A	Yes
Indiana	qPCR	*SMN1*	None	N/A	Yes
Kansas	qPCR	*SMN1*	Digital drop PCR	*SMN2*	Yes
Kentucky	qPCR	*SMN1*	None	N/A	Yes
Maine **	qPCR	*SMN1*	qPCR	*SMN1*exon 7 (Assay A) AND SMN1exon 7, *SMN1*intron 7 (Assay B) followed by sequencing as a third tier if all replicates show absent exon 7 (Assay A) and one or more replicates show present Exon 7 (Assay B)	No
Maryland	qPCR	*SMN1*	None	N/A	Yes
Massachusetts **	qPCR	*SMN1*	qPCR	*SMN1*exon 7 (Assay A) AND SMN1exon 7, *SMN1*intron 7 (Assay B) followed by sequencing as a third tier if all replicates show absent exon 7 (Assay A) and one or more replicates show present Exon 7 (Assay B)	No
Michigan	qPCR	*SMN1*	None	N/A	Yes
Minnesota	qPCR	*SMN1*	None	N/A	Yes
Mississippi	qPCR	*SMN1*	None	N/A	Yes
Missouri	qPCR	*SMN1*	None	N/A	Yes
Montana	qPCR	*SMN1*	Digital drop PCR	*SMN2*	Yes
Nebraska	qPCR	*SMN1*	None	N/A	Yes
New Hampshire **	qPCR	*SMN1*	qPCR	*SMN1*exon 7 (Assay A) AND SMN1exon 7, *SMN1*intron 7 (Assay B) followed by sequencing as a third tier if all replicates show absent exon 7 (Assay A) and one or more replicates show present Exon 7 (Assay B)	No
New York	qPCR	*SMN1*	Digital drop PCR	*SMN2*	Yes
North Carolina	qPCR	*SMN1*	None	N/A	Yes
Oklahoma	qPCR	*SMN1*	None	N/A	No
Pennsylvania	qPCR	*SMN1*	Multiplex Ligation PCR Amplification (MLPA)	*SMN2*	No
Rhode Island **	qPCR	*SMN1*	qPCR	*SMN1*exon 7 (Assay A) AND SMN1exon 7, *SMN1*intron 7 (Assay B) followed by sequencing as a third tier if all replicates show absent exon 7 (Assay A) and one or more replicates show present Exon 7 (Assay B)	No
Tennessee	qPCR	*SMN1*	Multiplex Ligation PCR Amplification (MLPA)	SMN2	Yes
Texas	qPCR	*SMN1*	RT-PCR	SMN2	Yes
Utah	qPCR	*SMN1*	None	N/A	Yes
Vermont **	qPCR	*SMN1*	qPCR	*SMN1*exon 7 (Assay A) AND SMN1exon 7, *SMN1*intron 7 (Assay B) followed by sequencing as a third tier if all replicates show absent exon 7 (Assay A) and one or more replicates show present Exon 7 (Assay B)	No
Washington	qPCR	*SMN1*	None	N/A	Yes
West Virginia	qPCR	*SMN1*	None	N/A	Yes
Wisconsin	qPCR	*SMN1*	Digital drop PCR	*SMN2*	Yes
Wyoming	qPCR	*SMN1*	None	N/A	Yes

* Not all states have a newborn screening laboratory; some newborn screening programs send their dried blood spot specimens to another laboratory. ^ While many states do not have a second-tier screen for SMA, many will perform a confirmation duplicate by using the same dried blood spot to repeat the qPCR for *SMN1* exon 7 deletion the following day. ** Massachusetts has developed, validated and implemented a three-tier, high-throughput algorithm for the detection of SMA-detected infants in Massachusetts, Maine, New Hampshire, Rhode Island, and Vermont [11].
